# The Antiquity and Evolutionary History of Social Behavior in Bees

**DOI:** 10.1371/journal.pone.0021086

**Published:** 2011-06-13

**Authors:** Sophie Cardinal, Bryan N. Danforth

**Affiliations:** Department of Entomology, Cornell University, Ithaca, New York, United States of America; Field Museum of Natural History, United States of America

## Abstract

A long-standing controversy in bee social evolution concerns whether highly eusocial behavior has evolved once or twice within the corbiculate Apidae. Corbiculate bees include the highly eusocial honey bees and stingless bees, the primitively eusocial bumble bees, and the predominantly solitary or communal orchid bees. Here we use a model-based approach to reconstruct the evolutionary history of eusociality and date the antiquity of eusocial behavior in apid bees, using a recent molecular phylogeny of the Apidae. We conclude that eusociality evolved once in the common ancestor of the corbiculate Apidae, advanced eusociality evolved independently in the honey and stingless bees, and that eusociality was lost in the orchid bees. Fossil-calibrated divergence time estimates reveal that eusociality first evolved at least 87 Mya (78 to 95 Mya) in the corbiculates, much earlier than in other groups of bees with less complex social behavior. These results provide a robust new evolutionary framework for studies of the organization and genetic basis of social behavior in honey bees and their relatives.

## Introduction

Eusociality, characterized by reproductive division of labor, cooperative brood care, and overlap of generations, is considered one of the key innovations that has allowed ants, bees, and termites to become the dominant organisms in terrestrial ecosystems [1]. Eusociality has arisen at least eight times in hymenopteran insects, and five of those origins are in bees [2]. However, uncertainty about tribal relationships within Apidae makes that exact number uncertain. One of the primary controversies in the evolution of sociality in bees lies within the corbiculates (Hymenoptera: Apidae), where a single versus dual-origin hypothesis for highly eusocial behavior has been extensively debated.

The corbiculate bees (a group of over 1000 species) are undoubtedly the most thoroughly studied of all bee lineages. The group is of particular interest because it includes advanced eusocial bees, the only bees to store harvestable honey, and the most important managed pollinators in agricultural settings (*i.e.*, the honey bee). In addition, corbiculate bees are model organisms for understanding the organization and evolution of social behavior in bees [3]–[7]. There are four extant monophyletic tribes: the highly eusocial Apini (honey bees) and Meliponini (stingless bees), the primitively eusocial Bombini (bumble bees), and the mostly solitary, communal, and weakly social Euglossini (orchid bees). The advanced eusocial Apini and Meliponini have morphologically distinct queens and workers with new nests founded by swarms [8], whereas the primitively eusocial Bombini have queens and workers that differ only in size, with new nests established by a single foundress. Non-parasitic orchid bees are usually referred to as being solitary or communal [9], but hints of more advanced forms of social behavior, including overlap of generations and cooperative brood care, have been reported in some taxa [10], [11].

While monophyly of the corbiculate bees as a whole is well supported and non-controversial [12], [13], the phylogeny of the corbiculate bee tribes has until recently remained remarkably unclear. In theory, there are 15 possible rooted trees for these four taxa, and some of the controversy arises from the fact that nine of these have been published as potential phylogenies (recently reviewed in [14]). Most morphological [12], [14]–[17], behavioral [18], and some combined morphological and molecular [19], [20] analyses support the phylogeny proposed by Michener [21]: (Euglossini+(Bombini+(Apini+Meliponini))). This phylogeny is consistent with a single origin of primitive eusociality (in the common ancestor of Bombini, Apini, and Meliponini) and a single origin of advanced eusociality (in the common ancestor of Apini and Meliponini) [8], [14], [15]. However, alternative phylogenies have been obtained based both on morphology [22] and molecular data [23]–[25]. Most molecular studies have supported the sister group relationship between Bombini and Meliponini, often with high bootstrap support, but with variable placement of Apini and Euglossini. This topology would imply two origins of advanced eusociality under simple parsimony reconstruction [22], [23], [25]. Earlier molecular studies have been criticized on various grounds, including poor outgroup sampling, poor choice of genes, and the possible impact of long-branch attraction [17], [26], [27]. The incongruence between the morphological and molecular results for corbiculates remains one of the most controversial aspects of apid phylogeny [20], [25], [28]. However, recent analysis of two large molecular datasets [13], [29] have addressed many of the limitations of previous molecular phylogenies and strongly support the phylogeny: ((Bombini+Meliponini)+(Apini+Euglossini)). We take this topology to be the best estimate of corbiculate relationships.

A well-supported molecular phylogeny based on extensive taxon sampling, including representatives of all apid subfamilies and tribes, allows us to use model-based methods to reconstruct the evolution of social behavior. Traditional parsimony methods are unable to distinguish between single or dual origins of eusociality when reconstructing social behavior on the molecular phylogeny. However, model-based approaches, such as maximum likelihood and Bayesian methods [30]–[35], may provide better insights into the evolutionary history of eusociality because they allow for uncertainty in tree topology, branch lengths, and relative rates of gains/losses to be incorporated into the reconstruction of ancestral states. Bayesian methods have been used to reconstruct ancestral states in social insects [36]–[38] but they have not been applied previously to the evolution of sociality in corbiculate bees. In this paper, we use Bayesian ancestral state reconstructions to elucidate the evolutionary history of eusocial behavior in apid bees using the molecular dataset of Cardinal *et al.* [13]. The results of our ancestral state reconstruction are then combined with the Cardinal *et al.* [13] fossil-calibrated chronogram to estimate the antiquity of eusociality in corbiculate bees. We then examine the antiquity of eusociality in corbiculates in relation to other eusocial insect lineages.

## Results

We ran our Bayesian ancestral state reconstructions using two different coding schemes. The results of the two coding schemes are largely congruent. The first scheme (which will be referred to as the *traditional scheme*) followed the behavioral state codings of previous studies on the evolution of social behavior within corbiculates [17], [19], [20], [28], whereas the second scheme (which will be referred to as the *complex scheme*) added an extra state representing Michener's [8] subsocial and parasocial levels of social organization among bees (Table S1)(see methods section for a more detailed explanation of the two different character state coding schemes). The complex coding scheme also incorporated directly into the analysis the wide range of social behaviors reported in orchid bees [10], [11], [39]–[42] and in the large and small carpenter bees (*Xylocopa* and *Ceratina*) (e.g. [43]–[47]).

**Figure 1:**
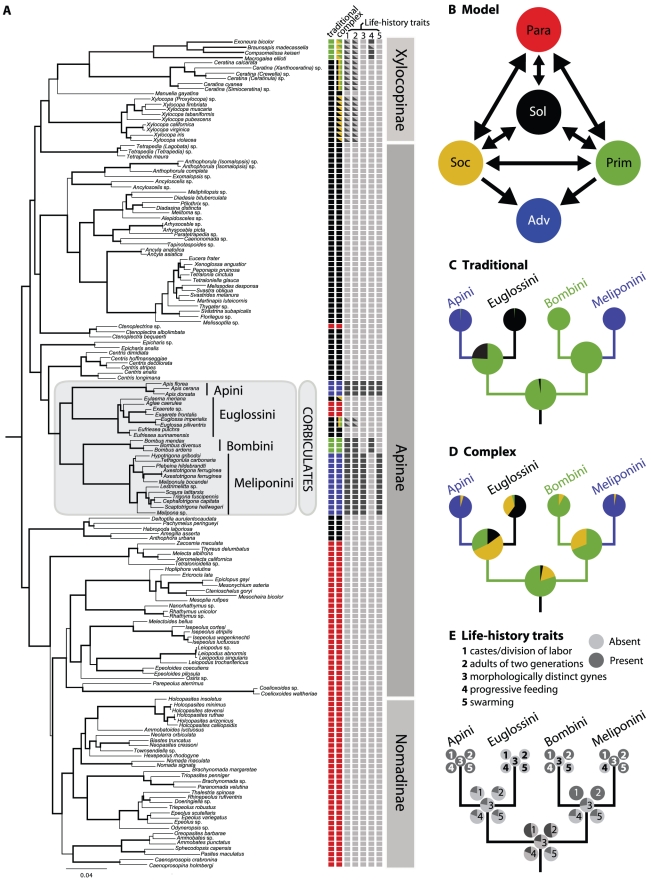
The evolution of eusociality in Apidae. a) The Bayesian maximum clade credibility tree of Apidae [13]. Posterior probabilities are represented by the thickness of the branches. Character state assignments of the taxa used for the ancestral state reconstruction of the traditional and complex social level character and of the 5 life-history traits are shown to the right of the tree (black = solitary, yellow = social, green = primitively eusocial, blue = advanced eusocial, red = parasitic, light grey = absent, dark grey = present). The character states do not necessarily represent the state of that particular species, but how that terminal taxon was coded to represent the state(s) of the clade it represents. b) Transitions allowed between the four behavioral states in our model-based ancestral state reconstruction of the complex social level character (Sol = solitary, Soc = social, Prim = primitively eusocial, Adv = advanced eusocial, and Paras = parasitic). The model was the same for the traditional behavioral character on level of sociality, but the state social was not included. c–e) Simplified version of the corbiculate phylogeny with pie charts representing the posterior probability of the ancestral state of the node for the c) traditional social level character, d) complex social level character , and e) five life-history traits.

Based on our model-based ancestral state reconstructions, the common ancestor of the corbiculates is estimated to be primitively eusocial (traditional: Posterior Probability (PP) = 98%, complex: PP = 79%) (Fig. 1c–d, Table S2). The common ancestor of Bombini+Meliponini is also reconstructed as primitively eusocial (traditional: PP = 100%, complex: PP = 68%), as is the common ancestor of Euglossini+Apini under the traditional coding (PP = 75%). Using the complex coding, the social state of the common ancestor of Euglossini+Apini is more ambiguous (53% PP of being social and 32% PP of being primitively eusocial). Allowing a non-zero rate of transition from solitary to advanced eusociality in the traditional analysis reconstructed the ancestral state of the corbiculates as being primitively eusocial with a PP of 86%. These results imply a single origin of eusocial behavior in the corbiculate bees with two independent origins of advanced eusocial behavior (in the stingless bees and honey bees), and a reversal from primitively eusocial (or social) to solitary/communal nesting in some orchid bees.

Results of the Bayes Factor tests support the hypothesis that the common ancestor of the corbiculates was primitively eusocial. We found strong support for a primitively eusocial common ancestor when compared with parasitic, social and advanced eusocial states (Table 1). The Bayes Factor comparing the likelihood of a primitively eusocial vs. solitary ancestor showed weaker, but positive, support for primitive eusociality as the ancestral state (Table 1). Collectively, the Bayes Factor tests corroborate the Bayesian reconstructions of a primitively eusocial corbiculate ancestor.

**Table 1:** Mean and Standard error (S.E.) of the Bayes Factor tests (n = 20) comparing the harmonic mean of the likelihoods of the Bayesian ancestral state reconstruction analyses with the common ancestor of corbiculates alternatively fixed as being solitary, social, primitively eusocial, advanced eusocial, and parasitic.

	Bayes Factor							
	Prim vs. Sol		Prim vs. Soc		Prim vs. Adv		Prim vs. Par	
Character	mean	S.E.	mean	S.E.	mean	S.E.	mean	S.E.
Traditional	3.45	0.79	NA	NA	159.71*	0.98	18.53*	0.64
Complex	4.73	0.91	7.28*	1.21	155.05*	0.99	19.15*	0.63

Sol: Solitary.

Prim: Primitively eusocial.

Soc: Social.

Adv: Advanced eusocial.

Par: Parasitic.

*Bayes Factor test strongly supports a primitively eusocial ancestor over the other state (i.e. Bayes Factor >5 [34]).

Breaking down the complex behavioral character representing the bees' levels of sociality into five simpler social life-history traits, also supports the conclusion of a primitively eusocial corbiculate ancestor. Model-based ancestral state reconstruction of these characters suggests that the corbiculate ancestor had colonies with adults of two-generations (PP = 53%), castes with division of labor (PP = 53%), mass provisioned offspring (PP = 81%), morphologically undifferentiated castes (PP = 78%), and establishment of new colonies by solitary females (PP = 81%) (Fig. 1e, Table S3).

**Figure 2:**
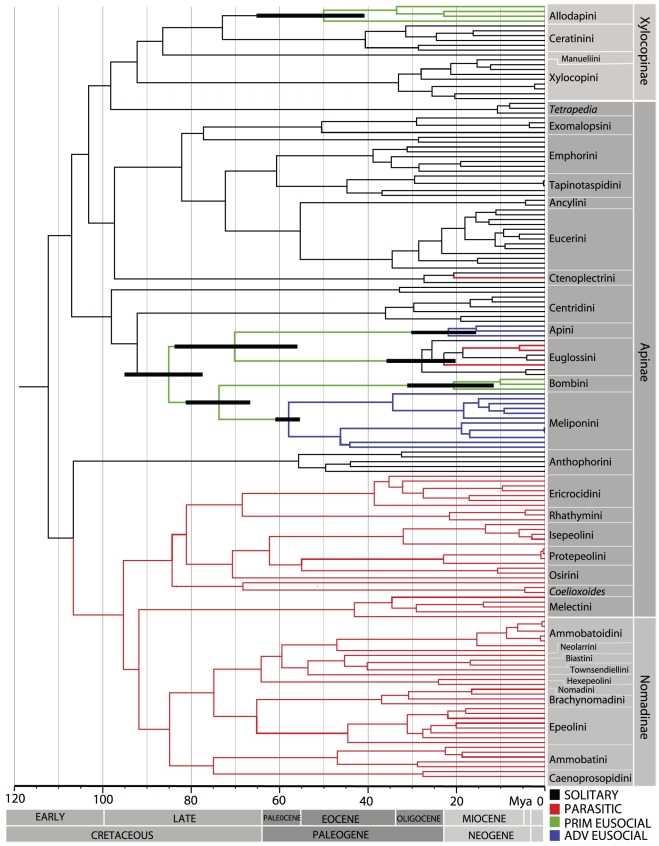
The antiquity of eusocial clades. The behavioral character is mapped onto the chronogram of Apidae [13] according to the results of the Bayesian ancestral state reconstruction of the traditional social level character. Outgroup taxa used in the fossil-calibrated phylogeny have been removed from the chronogram. Black bars represent the 95% highest posterior density (HPD) on the estimated age of the eusocial clades.

Mapping our behavioral character state reconstructions onto the chronogram of Cardinal *et al.* [13] (Fig. 2), we estimate that primitive eusociality evolved once in the allodapines, whose extant lineages originated 53 Mya (41 to 65 Mya), and once in the corbiculate bees, whose extant lineages originated 87 Mya (78 to 95 Mya). The estimated age of origin for extant members of the highly eusocial stingless bees is 58 Mya (56 to 61 Mya) and for extant members of the highly eusocial honey bees is 22 Mya (16 to 30 Mya). The primitively eusocial bumble bees are estimated to have originated 21 Mya (12 to 31 Mya) and orchid bees 28 Mya (21 to 35 Mya).

## Discussion

### Evolution of eusociality

According to our model-based ancestral state reconstructions, the ancestral state for corbiculate bees appears to be primitive eusociality (Fig. 1, Table S2). From this primitively eusocial ancestor, stingless bees and honey bees independently evolved advanced eusocial behavior. Our life-history traits analyses indicate that honey bees and stingless bees inherited castes with division of labor and colonies containing adults of two generations (characteristics of primitively eusocial colonies) from the common ancestor of corbiculates as a whole. However, they appear to have convergently evolved morphological differentiation between reproductive and worker castes and swarming behavior (characteristics of highly eusocial colonies). The evolution of advanced eusociality has only occured a handful of times, and thus it is particularly noteworthy that it has evolved twice within this one, relatively small, clade of bees.

While remarkable, a hypothesis of dual origins of advanced eusociality is congruent with early studies on corbiculate morphology [22] and social behavior [8]. Though Meliponini and Apini both have evolved elaborate social behavior, they differ substantially in the details of their social biology [8]. Both establish new colonies by swarming, but in stingless bees, it is a young queen that leaves to form a new nest whereas in honey bees it is the old queen that leaves the parental nest in search of a new nest site. This would therefore support the hypothesis that swarming is independently evolved in honey bees and stingless bees. The two tribes also differ in their mechanisms of recruitment to food sources. Stingless bees use social facilitation, odor trails and guides to lead bees to food sources. Honey bees communicate information about the location of food sources mostly through their well-studied dance language [48]. Apini and Meliponini also differ in how larvae are reared, queens are produced, gynes are killed, and in their nest architecture as summarized in [22]. These differences led Winston and Michener [22] to speculate that Meliponini and Apini independently evolved advanced eusociality, and that the common ancestor of the corbiculates might have had behavioral and morphological pre-adaptations for more advanced forms of social behavior (*i.e.*, a primitively eusocial common ancestor).

Our results help to reconcile detailed behavioral studies that have long hinted at the prevalence of eusocial behavior in orchid bees – behaviors that have previously been ignored in studies of corbiculate social evolution. While many authors have described Euglossini as solitary/communal [9], [49], [50], others have noted that some species (especially within *Euglossa*) have multiple-female associations in which some females forage and others guard the nest, suggestive of weak division of labor [10], [11], [39], [42], [51]–[53]. According to detailed studies on the nesting behavior of five different *Euglossa* species found in small colonies with semi-social and eusocial organization [10], [11], [40]–[42], multi–female nests may be formed by females of the same (sister-sister) or different (mother-daughter) generations, and the oldest female tends to be the dominant egg layer. Given our ancestral state reconstructions, one might interpret the weak reproductive division of labor in *Euglossa* as retention of the primitive eusocial state inferred to exist in the common ancestor of all corbiculates. We predict that further investigation should reveal additional evidence of more elaborate forms of sociality in orchid bees.

A reversal to solitary living in orchid bees has important implications for studies of the evolution of eusociality in corbiculates, because Euglossini is commonly taken to represent a retention of the “primitive” (ancestral) solitary condition. If Euglossini are indeed derived from an ancestor that was eusocial, then Euglossini do not represent a primitively solitary phenotype, but a secondarily solitary phenotype [54]. Using euglossines as representative of a “solitary corbiculate” in comparative studies is likely to lead to incorrect assessments of the genetics and behavior underlying the transition from the solitary mode of life, typical of the vast majority of bees, to the primitive and advanced modes of social organization evident in honey bees and their relatives. A more appropriate choice of a solitary bee group for comparison with corbiculate eusocial behavior would be bees in the genus *Centris*, which are truly solitary and strongly supported as the likely sister group to the corbiculates [13].

Results of a recent study [55] investigating the genetic changes involved in the evolution of eusociality may be interpreted differently in light of our hypothesis that all four extant tribes of corbiculate bees shared a common, primitively eusocial common ancestor. Woodard *et al.* [55] analyzed their data under the assumption that Bombini+Meliponini and Apini represent two independent origins of eusociality, while our results suggest only one (Fig. 1). Furthermore, the orchid bee species that were used as representatives of solitary bees have both been shown to exhibit social behavior. Cooperative cell construction and provisioning has been reported in *Eulaema nigrita* [39], and nests with multiple females of different generations showing reproductive division of labor have been reported in *Euglossa cordata* [40]. Consequently, genes identified by Woodard *et al.* [55] to have convergently evolved an accelerated rate of amino acid substitution in the clade Bombini+Meliponini and the Apini may in fact be due to common ancestry instead of convergence. We would expect however those genes identified to have convergently evolved an accelerated rate of evolution in the highly eusocial Apini and Meliponini to hold true under our hypothesis of the evolutionary history of eusociality.

### Antiquity of eusociality

The oldest reported fossil of a eusocial bee is *Cretotrigona prisca*, a stingless bee found in the Late Cretaceous amber of New Jersey [56]. The age of this fossil is uncertain, with estimates on its origin ranging from the Paleocene [57] to the Late Cretaceous [56], [58]. Although this uncertainty persists, the fossil is now considered to be of Late Maastrichtian age (ca. 65–70 Ma) [59]. There are also numerous corbiculate fossils from the Baltic amber which is ∼44 My old [60]. Many of these fossil taxa do not fall within extant tribes, but have instead been assigned to their own fossil tribes. The exact phylogenetic affinities of these extinct tribes to the extant corbiculates is unclear, but in a morphological cladistic analysis including both the extinct and extant corbiculates, the extinct tribes Melikertini, Electrapini and Electrobombini formed a monophyletic group with Apini and Meliponini [16]. Although the social state of the fossil taxa cannot be directly observed, the presence of a reduced metasoma in some fossils supports a hypothesis that these fossilized specimens represent the worker caste of highly eusocial species [16]. Also, a few of the fossilized bees appear to have microscopic barbs on the sting, a characteristic associated with some workers of eusocial species. Therefore, fossil evidence suggests that eusocial corbiculates were present at least 44 Mya and most likely 65 Mya (depending on the true age of the *Cretotrigona prisca* fossil).

To further refine our estimates of the antiquity of eusociality, we combined information from the fossil record with our hypothesis of the apid phylogeny based on molecular data and our hypothesis on the evolutionary history of eusociality. Relaxed fossil calibrated molecular clock models have been used to estimate the age of Euglossini (27–42 Mya [61]), Bombini (25 to 40 Mya [62]), Meliponini (81 to 96 Mya [63]), Apini (28 to 36 [64]), corbiculates (81to 94 Mya [64]) and Allodapini (39–69 Mya) [65]). We based our estimates of the ages of the different eusocial clades on the cladogram of Cardinal *et al.* [13] (Fig. 2) which included the most calibration points due to the larger taxonomic focus of the study. The range of the estimated ages for the four extant corbiculate tribes from this study overlapped with the age range estimates from the previous analyses with the exception of the age estimates for Meliponini. The Cardinal *et al.* [13] age estimate of 58 Mya (56 to 61 Mya) for the common ancestor of extant Meliponini suggests that *Cretotrigona prisca* represents a stem lineage of Meliponini or that the younger estimates [57] for the age of the fossil are more accurate. Rasmussen *et al.* [63] and Ramírez *et al.* [64] did not explore ages younger than 65 Mya in their analyses based on the belief that the *Cretotrigona prisca* fossil is at least 65 My old and not a stem lineage of Meliponini.

If any of the extinct fossil taxa, which may have been eusocial, are later found to be sister to all of the extant corbiculates, then the evolution of eusociality would predate the common ancestor of the extant corbiculates, which we estimate to have evolved 87 Mya (78 to 95 Mya). An accurate phylogenetic placement of these fossil taxa could also change our interpretation of the evolutionary history of eusociality within corbiculates as could the discovery of new fossil taxa.

**Figure 3:**
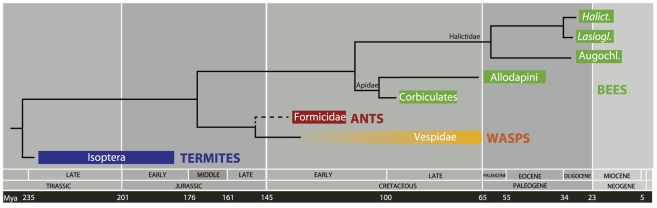
Estimated ages of the major eusocial insect clades. Colored boxes indicate the time period in which the eusocial members of the clade are estimated to have originated. All estimates are based on fossil calibrated divergence time analyses except for the eusocial Vespidae, which is based on the fossil of a polistine nest from the Late Cretaceous [66]. This fossil provides a minimum age for the origin of eusocial wasps which probably originated sometime in the mid-Cretaceous [67]. Molecular studies, however, have indicated that the eusocial vespids do not form a monophyletic group and instead represent two independent eusocial lineages [84]. There is also uncertainty in the phylogenetic placement of ants relative to bees and vespids. The traditional relationships are depicted here, with ants more closely related to vespids than to bees [85], although a more recent molecular analysis suggests that ants share a more recent common ancestor with bees than vespids [86]. Termites are estimated to have originated sometime between 180 and 230 Mya [70], ants between 115 and 135 Mya [69], corbiculates 87 Mya (78 to 95 Mya), allodapines 53 Mya (41 to 65 Mya), eusocial *Halictus* (*Halict.*) 21 Mya (15 to 28 Mya) [71], eusocial *Lasioglossum* (*Lasiogl.*) 22 Mya (15 to 29 Mya) [71], and eusocial Augochlorini (Augochl.) 20 Mya (12 to 29 Mya) [71].

Fossil calibrated phylogenies have now been used to date the antiquity of eusociality in all five clades of eusocial bees. While eusocial wasps [66], [67], and ants [68], [69]show origins well within the Cretaceous (65–140 Ma), and termites are estimated to have originated sometime between 180 and 230 Mya [70], bees show independent origins of eusocial lineages over a broad timescale from late Cretaceous (87 Ma) to the Miocene (20 Ma) [13], [61]–[65], [71] (Fig. 3). Our results indicate that eusocial complexity within bees is roughly correlated with age, such that more ancient lineages (corbiculates) show more complex social organization than the more recent groups (allodapines and halictines). Not only do more recent lineages show less complex forms of social organization, they are also more prone to showing reversals from eusociality to solitary nesting. Danforth [72] documented repeated reversals to solitary nesting in each of the three eusocial halictine clades, which arose relatively recently, whereas reversals have not been observed in older lineages such as ants and allodapine bees [73]. Our hypothesis of a reversal to solitary nesting in Euglossini is possibly the oldest reversal to solitary nesting reported for bees, although the reversal does appear to have occurred from a primitively eusocial ancestor. We estimate that complex social behaviors characteristic of honey bees and stingless bees, such as caste polymorphism, complex forms of communication, elaborate nest architecture, and age polyethism, have evolved over an 80 million year timespan.

## Materials and Methods

We used a random sample of 10 000 post-burnin tree topologies (including branch length data) from the Cardinal *et al.* [13] Bayesian phylogenetic analysis to run a number of ancestral state reconstructions using the program BayesTraits v.1.0 [34]. The broad taxon sampling used in this study is better suited for ancestral state reconstruction of social behavior within corbiculates than the Kawakita *et al.* [29] or the Woodard *et al.* [55] study because it allows for more accurate estimates of transition rates between states. We first coded all terminal taxa for a general behavioral typological character representing the social level of the bees (see Table S1 for the defining life-history traits of each social level). Following Michener [8], we coded the orchid bees as being solitary/communal, the bumble bees as being primitively eusocial and the honey bees and stingless bees as being highly eusocial (see Fig. 1a), as previous authors have done (e.g. [17], [19], [20], [28]). Because of our broader taxon sampling, we added a fourth state to accommodate the cleptoparasitic taxa included in our study. Although this coding represents the traditional view of sociality within corbiculates and other apid bees, reports of cooperative cell construction and provisioning in *Eulaema nigrita* [39], nests with associations of females of different generations with the oldest bee usually being the dominant egg layer in *Euglossa fimbriata*, *E. cordata*, *E. atroveneta*, *E. townsendi*, *and E. viridissima* [10], [11], [40]–[42] indicate that this is an oversimplification. To incorporate directly into the analysis the wide range of social behaviors found in orchid bees, we coded a second behavioral character in which we added a fifth state (social) representing Michener's [8] subsocial and parasocial levels of social organization among bees. This allowed us to differentiate taxa that have social tendencies (e.g. colonies with adults of two-generations during the breeding season or evidence of division of labor) from strictly solitary/communal taxa. Adding this fifth state also allowed us to incorporate reports of sociality within *Xylocopa* and *Ceratina* (e.g. [43]–[47]).

Most *Xylocopa* colonies consist of a foraging egg layer and a non-foraging guard (usually mother and daughter). The egg layer feeds nectar to other adults in the nest while she makes and provisions cells. The guard bee cleans and guards the nest while the dominant egg layer is foraging. We coded *Xylocopa* as being either solitary or social but not eusocial because these castes are considered ontogenetic stages rather than classes of individuals with the guard bee representing more of an inactive stage while waiting to take over the nest or establish a new nest [45], [46]. Daughters eventually mate and forage for their own brood. However, there have been reports of females usurping the nest of another bee which sometimes stays in the nest and becomes the usurper's guard [74], [75]. Also, the finding of worn uninseminated females of *Xylocopa sonorina* suggests the possibility that some individuals may be permanent workers [45]. In socially nesting *Ceratina*, division of labor is often found, but with the dominant egg layer staying in the nest while the subordinate bee forages and feeds the dominant bee [43], [45], [47]. Unlike in *Xylocopa*, the workers are generally not thought to be a developmental stage leading to future reproductive activity [45]. We therefore coded *Ceratina* as being solitary, social or primitively eusocial. We also coded the allodapines as being social or primitively eusocial based on information in the literature [45], [76]–[81].

For both versions of the behavioral character (traditional and complex), we ran the MCMC analysis with most transition rate priors having a uniform distribution with a range from 0 to 100. This assumes that all values of the parameters are equally likely *a priori* and therefore limits the assumptions being made. We set the prior probability on transitions from parasitic or solitary nesting to advanced eusociality to zero (Fig. 1b) because we considered it highly unlikely that a bee could evolve from being parasitic or solitary to having morphologically distinct castes and swarm founding without an intermediate step. Relaxation of this prior did not substantially alter our results. We also did not allow transitions from advanced eusocial behavior to any other state because queens in advanced eusocial species cannot forage or found new nests independent of workers [1], [8], [22]. We ran both types of analyses five times for 100 million generations and observed the trace files of the model parameters to discard all generations prior to the runs reaching stability as burn-in.

In order to statistically test whether there was significant support for a primitively eusocial common ancestor, we ran the traditional and complex Bayesian ancestral state reconstruction analyses 20 times with the common ancestor alternatively fixed as solitary, parasitic, primitively eusocial and advanced eusocial. We then conducted pairwise comparisons of the harmonic mean of the likelihoods for each of the 20 replicates under each of these models using a Bayes Factor test [34].

To further investigate what level of sociality the common ancestor of the corbiculate bees had, we broke down our complex behavioral character into 5 social life-history traits described in Table S1. We followed the methods described above, but applied a reversible jump model with priors obtained from a hyperprior approach with an exponential distribution seeded from a uniform on the interval 0 to 10. We were able to apply a reversible jump model for these analyses because we did not need to place any constraints on any of the character state transition rates. We ran each analysis 5 times for 100 million generations discarding the appropriate number of generations as burn-in.

To date the antiquity of eusocial lineages in apid bees, we mapped our behavioral character state reconstructions onto the chronogram of Cardinal *et al.* [13]. The chronogram was constructed by using a fossil calibrated uncorrelated relaxed molecular clock model [82] in the program BEAST v1.4.8 [83], sequence data from seven genes for 190 bees, and 10 calibration points with prior age estimates based on paleontological evidence. The rate at each branch was drawn from an underlying log-normal distribution. Uncertainty in the age of the calibration points was incorporated into the analysis by assuming that the probability of the node being a certain age follows a lognormal distribution with a rigid minimum bound. This allows us to assume that the actual divergence event took place some time prior to the earliest appearance of fossil evidence, but that the age of the node is more likely to be close to the age of the oldest known fossil, and less likely to be significantly older. More details on the chronogram and information on each calibration point are given in Cardinal *et al.* [13]. Here we present the 95% highest posterior density (HPD) on the estimated age of the eusocial clades when the root node age of bees was set to 120 Mya and the analysis was run for 200 million generations. Corbiculates were always estimated to be of Late Cretaceous origin even when the root node age of bees ranged from 90 Mya to 145 Mya.

## Supporting Information

Table S1 **Social life-history traits of various levels of social organization found in bees, following Michener** [**8**] **.**
(DOC)

Table S2 **Posterior probability (Mean and Standard error) of the ancestral states of seven key nodes from the Bayesian ancestral state reconstructions (5 replicates) of the traditional (top) and complex (bottom) coding scheme.**
(DOC)

Table S3 **Posterior probability (Mean and Standard error) of the ancestral states of seven key nodes from the Bayesian ancestral state reconstructions (5 replicates) of the five life-history (LH) traits.**
(DOC)
