# Supplementary material for: The Antiquity and Evolutionary History of Social Behavior in Bees
Source: PLoS One. 2011 Jun 13;6(6):e21086. doi: 10.1371/journal.pone.0021086 (PMC3113908; doi:10.1371/journal.pone.0021086)
Supplement: Table S1 — Social life-history traits of various levels of social organization found in bees, following Michener [8] . (DOC) [file pone.0021086.s001.doc]

**Table S1.** Social life-history traits of various levels of social organization found in bees, following Michener [8].

| **Level** | **Castes^*^** | **Gen. overlap^**^** | **Morph. diff.^†^** | **Prog. feed.^‡^** | **Swarming^^** |
| --- | --- | --- | --- | --- | --- |
| Solitary | - | - | - | - | - |
| Social^♦^ | +/- | +/- | - | +/- | - |
| Prim. eusocial | + | + | - | +/- | - |
| High. eusocial | + | + | + | +/- | + |

+ indicates that column heading applies, - indicates that it does not, +/- indicates variability.

^*^Castes and division of labor

^**^Colonies with adults of two-generations (matrifilial)

^†^Female castes are morphologically different; gynes (if any) cannot survive alone

^‡^Progressive feeding

^^New colonies established by swarming.

^♦^Social colonies may have castes and division of labor or colonies with adults of two-generations during the active breeding season, but not both as primitively eusocial (Prim. eusocial) and highly eusocial (High. eusocial) colonies do.
