# Supplementary material for: The Antiquity and Evolutionary History of Social Behavior in Bees
Source: PLoS One. 2011 Jun 13;6(6):e21086. doi: 10.1371/journal.pone.0021086 (PMC3113908; doi:10.1371/journal.pone.0021086)
Supplement: Table S2 — Posterior probability (Mean and Standard error) of the ancestral states of seven key nodes from the Bayesian ancestral state reconstructions (5 replicates) of the traditional (top) and complex (bottom) coding scheme. (DOC) [file pone.0021086.s002.doc]

**Table S2.** Probability (Mean and Standard error) of the ancestral states of seven key nodes from the Bayesian ancestral state reconstructions (5 replicates) of the traditional (top) and complex (bottom) coding scheme.

| **Character** | **Rep.** | **Corbiculates** | | | | | **Bom_Meli^*^** | | | | | **Api_Eugl^**^** | | | | | **Euglossini** | | | | | **Bombini** | | | | | **Apini** | | | | | **Meliponini** | | | | |
| --- | --- | --- | --- | --- | --- | --- | --- | --- | --- | --- | --- | --- | --- | --- | --- | --- | --- | --- | --- | --- | --- | --- | --- | --- | --- | --- | --- | --- | --- | --- | --- | --- | --- | --- | --- | --- |
|  |  | **Sol** | **Soc** | **Prim** | **Adv** | **Para** | **Sol** | **Soc** | **Prim** | **Adv** | **Para** | **Sol** | **Soc** | **Prim** | **Adv** | **Para** | **Sol** | **Soc** | **Prim** | **Adv** | **Para** | **Sol** | **Soc** | **Prim** | **Adv** | **Para** | **Sol** | **Soc** | **Prim** | **Adv** | **Para** | **Sol** | **Soc** | **Prim** | **Adv** | **Para** |
| **Traditional** |  |  |  |  |  |  |  |  |  |  |  |  |  |  |  |  |  |  |  |  |  |  |  |  |  |  |  |  |  |  |  |  |  |  |  |  |
|  | 1 | 0.02 | NA | 0.98 | 0.00 | 0.00 | 0.00 | NA | 1.00 | 0.00 | 0.00 | 0.25 | NA | 0.75 | 0.00 | 0.00 | 0.99 | NA | 0.01 | 0.00 | 0.00 | 0.00 | NA | 1.00 | 0.00 | 0.00 | 0.00 | NA | 0.01 | 0.99 | 0.00 | 0.00 | NA | 0.00 | 1.00 | 0.00 |
|  | 2 | 0.02 | NA | 0.98 | 0.00 | 0.00 | 0.00 | NA | 1.00 | 0.00 | 0.00 | 0.24 | NA | 0.75 | 0.00 | 0.00 | 0.99 | NA | 0.01 | 0.00 | 0.00 | 0.00 | NA | 1.00 | 0.00 | 0.00 | 0.00 | NA | 0.01 | 0.99 | 0.00 | 0.00 | NA | 0.00 | 1.00 | 0.00 |
|  | 3 | 0.02 | NA | 0.98 | 0.00 | 0.00 | 0.00 | NA | 1.00 | 0.00 | 0.00 | 0.24 | NA | 0.75 | 0.00 | 0.00 | 0.99 | NA | 0.01 | 0.00 | 0.00 | 0.00 | NA | 1.00 | 0.00 | 0.00 | 0.00 | NA | 0.01 | 0.99 | 0.00 | 0.00 | NA | 0.00 | 1.00 | 0.00 |
|  | 4 | 0.02 | NA | 0.98 | 0.00 | 0.00 | 0.00 | NA | 1.00 | 0.00 | 0.00 | 0.24 | NA | 0.75 | 0.00 | 0.00 | 0.99 | NA | 0.01 | 0.00 | 0.00 | 0.00 | NA | 1.00 | 0.00 | 0.00 | 0.00 | NA | 0.01 | 0.99 | 0.00 | 0.00 | NA | 0.00 | 1.00 | 0.00 |
|  | 5 | 0.02 | NA | 0.98 | 0.00 | 0.00 | 0.00 | NA | 1.00 | 0.00 | 0.00 | 0.24 | NA | 0.75 | 0.00 | 0.00 | 0.99 | NA | 0.01 | 0.00 | 0.00 | 0.00 | NA | 1.00 | 0.00 | 0.00 | 0.00 | NA | 0.01 | 0.99 | 0.00 | 0.00 | NA | 0.00 | 1.00 | 0.00 |
|  | **Mean** | **0.02** |  | **0.98** | **0.00** | **0.00** | **0.00** |  | **1.00** | **0.00** | **0.00** | **0.24** |  | **0.75** | **0.00** | **0.00** | **0.99** |  | **0.01** | **0.00** | **0.00** | **0.00** |  | **1.00** | **0.00** | **0.00** | **0.00** |  | **0.01** | **0.99** | **0.00** | **0.00** |  | **0.00** | **1.00** | **0.00** |
|  | **S.E.** | **0.00** |  | **0.00** | **0.00** | **0.00** | **0.00** |  | **0.00** | **0.00** | **0.00** | **0.00** |  | **0.00** | **0.00** | **0.00** | **0.00** |  | **0.00** | **0.00** | **0.00** | **0.00** |  | **0.00** | **0.00** | **0.00** | **0.00** |  | **0.00** | **0.00** | **0.00** | **0.00** |  | **0.00** | **0.00** | **0.00** |
| **Complex** |  |  |  |  |  |  |  |  |  |  |  |  |  |  |  |  |  |  |  |  |  |  |  |  |  |  |  |  |  |  |  |  |  |  |  |  |
|  | 1 | 0.03 | 0.18 | 0.78 | 0.00 | 0.00 | 0.01 | 0.31 | 0.68 | 0.00 | 0.00 | 0.15 | 0.53 | 0.32 | 0.00 | 0.00 | 0.59 | 0.37 | 0.04 | 0.00 | 0.00 | 0.00 | 0.08 | 0.91 | 0.00 | 0.00 | 0.00 | 0.04 | 0.00 | 0.95 | 0.00 | 0.00 | 0.03 | 0.00 | 0.97 | 0.00 |
|  | 2 | 0.03 | 0.18 | 0.79 | 0.00 | 0.00 | 0.01 | 0.31 | 0.68 | 0.00 | 0.00 | 0.15 | 0.52 | 0.32 | 0.00 | 0.01 | 0.60 | 0.36 | 0.04 | 0.00 | 0.00 | 0.00 | 0.08 | 0.92 | 0.00 | 0.00 | 0.00 | 0.04 | 0.00 | 0.95 | 0.00 | 0.00 | 0.03 | 0.00 | 0.97 | 0.00 |
|  | 3 | 0.03 | 0.18 | 0.78 | 0.00 | 0.00 | 0.01 | 0.31 | 0.68 | 0.00 | 0.00 | 0.15 | 0.53 | 0.32 | 0.00 | 0.00 | 0.61 | 0.35 | 0.04 | 0.00 | 0.00 | 0.00 | 0.08 | 0.92 | 0.00 | 0.00 | 0.00 | 0.04 | 0.00 | 0.95 | 0.00 | 0.00 | 0.03 | 0.00 | 0.97 | 0.00 |
|  | 4 | 0.03 | 0.17 | 0.80 | 0.00 | 0.00 | 0.00 | 0.31 | 0.69 | 0.00 | 0.00 | 0.15 | 0.53 | 0.32 | 0.00 | 0.01 | 0.60 | 0.36 | 0.04 | 0.00 | 0.00 | 0.00 | 0.08 | 0.92 | 0.00 | 0.00 | 0.00 | 0.04 | 0.00 | 0.95 | 0.00 | 0.00 | 0.03 | 0.00 | 0.97 | 0.00 |
|  | 5 | 0.03 | 0.18 | 0.79 | 0.00 | 0.00 | 0.00 | 0.31 | 0.68 | 0.00 | 0.00 | 0.15 | 0.53 | 0.32 | 0.00 | 0.00 | 0.59 | 0.37 | 0.04 | 0.00 | 0.00 | 0.00 | 0.08 | 0.92 | 0.00 | 0.00 | 0.00 | 0.04 | 0.00 | 0.95 | 0.00 | 0.00 | 0.03 | 0.00 | 0.97 | 0.00 |
|  | **Mean** | **0.03** | **0.18** | **0.79** | **0.00** | **0.00** | **0.01** | **0.31** | **0.68** | **0.00** | **0.00** | **0.15** | **0.53** | **0.32** | **0.00** | **0.00** | **0.60** | **0.36** | **0.04** | **0.00** | **0.00** | **0.00** | **0.08** | **0.92** | **0.00** | **0.00** | **0.00** | **0.04** | **0.00** | **0.95** | **0.00** | **0.00** | **0.03** | **0.00** | **0.97** | **0.00** |
|  | **S.E.** | **0.00** | **0.00** | **0.00** | **0.00** | **0.00** | **0.00** | **0.00** | **0.00** | **0.00** | **0.00** | **0.00** | **0.00** | **0.00** | **0.00** | **0.00** | **0.00** | **0.00** | **0.00** | **0.00** | **0.00** | **0.00** | **0.00** | **0.00** | **0.00** | **0.00** | **0.00** | **0.00** | **0.00** | **0.00** | **0.00** | **0.00** | **0.00** | **0.00** | **0.00** | **0.00** |

Sol: Solitary

Soc: Social

Prim: Primitively eusocial

Adv: Advanced eusocial

Para: Parasitic

NA: Not applicable

S.E.: Standard error

^*^common ancestor of Bombini and Meliponini

^**^common ancestor of Apini and Euglossini
