# Supplementary material for: The Antiquity and Evolutionary History of Social Behavior in Bees
Source: PLoS One. 2011 Jun 13;6(6):e21086. doi: 10.1371/journal.pone.0021086 (PMC3113908; doi:10.1371/journal.pone.0021086)
Supplement: Table S3 — Posterior probability (Mean and Standard error) of the ancestral states of seven key nodes from the Bayesian ancestral state reconstructions (5 replicates) of the five life-history (LH) traits. (DOC) [file pone.0021086.s003.doc]

**Table S3.** Posterior probability (Mean and Standard error) of the ancestral states of seven key nodes from the Bayesian ancestral state reconstructions (5 replicates) of the five life-history (LH) traits.

| **LH traits** | **Rep** | **Corbiculates** | | **Bom_Meli^*^** | | **Api_Eugl^**^** | | **Euglossini** | | **Bombini** | | **Apini** | | **Meliponini** | |
| --- | --- | --- | --- | --- | --- | --- | --- | --- | --- | --- | --- | --- | --- | --- | --- |
|  |  | **Abs** | **Pres** | **Abs** | **Pres** | **Abs** | **Pres** | **Abs** | **Pres** | **Abs** | **Pres** | **Abs** | **Pres** | **Abs** | **Pres** |
| **Castes^†^** | 1 | 0.47 | 0.53 | 0.00 | 1.00 | 0.79 | 0.21 | 1.00 | 0.00 | 0.00 | 1.00 | 0.00 | 1.00 | 0.00 | 1.00 |
|  | 2 | 0.48 | 0.52 | 0.00 | 1.00 | 0.79 | 0.21 | 1.00 | 0.00 | 0.00 | 1.00 | 0.00 | 1.00 | 0.00 | 1.00 |
|  | 3 | 0.47 | 0.53 | 0.00 | 1.00 | 0.79 | 0.21 | 1.00 | 0.00 | 0.00 | 1.00 | 0.00 | 1.00 | 0.00 | 1.00 |
|  | 4 | 0.47 | 0.53 | 0.00 | 1.00 | 0.79 | 0.21 | 1.00 | 0.00 | 0.00 | 1.00 | 0.00 | 1.00 | 0.00 | 1.00 |
|  | 5 | 0.47 | 0.53 | 0.00 | 1.00 | 0.79 | 0.21 | 1.00 | 0.00 | 0.00 | 1.00 | 0.00 | 1.00 | 0.00 | 1.00 |
|  | **Mean** | **0.47** | **0.53** | **0.00** | **1.00** | **0.79** | **0.21** | **1.00** | **0.00** | **0.00** | **1.00** | **0.00** | **1.00** | **0.00** | **1.00** |
|  | **S.E.** | **0.00** | **0.00** | **0.00** | **0.00** | **0.00** | **0.00** | **0.00** | **0.00** | **0.00** | **0.00** | **0.00** | **0.00** | **0.00** | **0.00** |
| **Gen.over^‡^** | 1 | 0.46 | 0.54 | 0.00 | 1.00 | 0.79 | 0.21 | 1.00 | 0.00 | 0.00 | 1.00 | 0.00 | 1.00 | 0.00 | 1.00 |
|  | 2 | 0.47 | 0.53 | 0.00 | 1.00 | 0.79 | 0.21 | 1.00 | 0.00 | 0.00 | 1.00 | 0.00 | 1.00 | 0.00 | 1.00 |
|  | 3 | 0.48 | 0.52 | 0.00 | 1.00 | 0.79 | 0.21 | 1.00 | 0.00 | 0.00 | 1.00 | 0.00 | 1.00 | 0.00 | 1.00 |
|  | 4 | 0.48 | 0.52 | 0.00 | 1.00 | 0.79 | 0.21 | 1.00 | 0.00 | 0.00 | 1.00 | 0.00 | 1.00 | 0.00 | 1.00 |
|  | 5 | 0.47 | 0.53 | 0.00 | 1.00 | 0.79 | 0.21 | 1.00 | 0.00 | 0.00 | 1.00 | 0.00 | 1.00 | 0.00 | 1.00 |
|  | **Mean** | **0.47** | **0.53** | **0.00** | **1.00** | **0.79** | **0.21** | **1.00** | **0.00** | **0.00** | **1.00** | **0.00** | **1.00** | **0.00** | **1.00** |
|  | **S.E.** | **0.00** | **0.00** | **0.00** | **0.00** | **0.00** | **0.00** | **0.00** | **0.00** | **0.00** | **0.00** | **0.00** | **0.00** | **0.00** | **0.00** |
| **Morph.diff.^^** | 1 | 0.81 | 0.19 | 0.76 | 0.24 | 0.81 | 0.19 | 1.00 | 0.00 | 1.00 | 0.00 | 0.00 | 1.00 | 0.00 | 1.00 |
|  | 2 | 0.81 | 0.19 | 0.75 | 0.25 | 0.81 | 0.19 | 1.00 | 0.00 | 1.00 | 0.00 | 0.00 | 1.00 | 0.00 | 1.00 |
|  | 3 | 0.81 | 0.19 | 0.76 | 0.24 | 0.81 | 0.19 | 1.00 | 0.00 | 1.00 | 0.00 | 0.00 | 1.00 | 0.00 | 1.00 |
|  | 4 | 0.65 | 0.35 | 0.60 | 0.40 | 0.65 | 0.35 | 1.00 | 0.00 | 0.99 | 0.01 | 0.00 | 1.00 | 0.00 | 1.00 |
|  | 5 | 0.81 | 0.19 | 0.76 | 0.24 | 0.81 | 0.19 | 1.00 | 0.00 | 1.00 | 0.00 | 0.00 | 1.00 | 0.00 | 1.00 |
|  | **Mean** | **0.78** | **0.22** | **0.73** | **0.27** | **0.78** | **0.22** | **1.00** | **0.00** | **1.00** | **0.00** | **0.00** | **1.00** | **0.00** | **1.00** |
|  | **S.E.** | **0.03** | **0.03** | **0.03** | **0.03** | **0.03** | **0.03** | **0.00** | **0.00** | **0.00** | **0.00** | **0.00** | **0.00** | **0.00** | **0.00** |
| **Prog.feed.^^** | 1 | 0.81 | 0.19 | 0.76 | 0.24 | 0.81 | 0.19 | 1.00 | 0.00 | 0.00 | 1.00 | 0.00 | 1.00 | 1.00 | 0.00 |
|  | 2 | 0.82 | 0.18 | 0.76 | 0.24 | 0.81 | 0.19 | 1.00 | 0.00 | 0.00 | 1.00 | 0.00 | 1.00 | 1.00 | 0.00 |
|  | 3 | 0.81 | 0.19 | 0.76 | 0.24 | 0.81 | 0.19 | 1.00 | 0.00 | 0.00 | 1.00 | 0.00 | 1.00 | 1.00 | 0.00 |
|  | 4 | 0.81 | 0.19 | 0.76 | 0.24 | 0.81 | 0.19 | 1.00 | 0.00 | 0.00 | 1.00 | 0.00 | 1.00 | 1.00 | 0.00 |
|  | 5 | 0.82 | 0.18 | 0.76 | 0.24 | 0.82 | 0.18 | 1.00 | 0.00 | 0.00 | 1.00 | 0.00 | 1.00 | 1.00 | 0.00 |
|  | **Mean** | **0.81** | **0.19** | **0.76** | **0.24** | **0.81** | **0.19** | **1.00** | **0.00** | **0.00** | **1.00** | **0.00** | **1.00** | **1.00** | **0.00** |
|  | **S.E.** | **0.00** | **0.00** | **0.00** | **0.00** | **0.00** | **0.00** | **0.00** | **0.00** | **0.00** | **0.00** | **0.00** | **0.00** | **0.00** | **0.00** |
| **Swarming^♦^** | 1 | 0.81 | 0.19 | 0.76 | 0.24 | 0.81 | 0.19 | 1.00 | 0.00 | 1.00 | 0.00 | 0.00 | 1.00 | 0.00 | 1.00 |
|  | 2 | 0.81 | 0.19 | 0.75 | 0.25 | 0.81 | 0.19 | 1.00 | 0.00 | 1.00 | 0.00 | 0.00 | 1.00 | 0.00 | 1.00 |
|  | 3 | 0.81 | 0.19 | 0.76 | 0.24 | 0.81 | 0.19 | 1.00 | 0.00 | 1.00 | 0.00 | 0.00 | 1.00 | 0.00 | 1.00 |
|  | 4 | 0.81 | 0.19 | 0.76 | 0.24 | 0.81 | 0.19 | 1.00 | 0.00 | 1.00 | 0.00 | 0.00 | 1.00 | 0.00 | 1.00 |
|  | 5 | 0.81 | 0.19 | 0.75 | 0.25 | 0.81 | 0.19 | 1.00 | 0.00 | 1.00 | 0.00 | 0.00 | 1.00 | 0.00 | 1.00 |
|  | **Mean** | **0.81** | **0.19** | **0.76** | **0.24** | **0.81** | **0.19** | **1.00** | **0.00** | **1.00** | **0.00** | **0.00** | **1.00** | **0.00** | **1.00** |
|  | **S.E.** | **0.00** | **0.00** | **0.00** | **0.00** | **0.00** | **0.00** | **0.00** | **0.00** | **0.00** | **0.00** | **0.00** | **0.00** | **0.00** | **0.00** |

Abs :Absent

Pres: Present

Rep: Replicate

**^*^**Common ancestor of Bombini and Meliponini

**^**^**Common ancestor of Apini and Euglossini

**^†^**Castes and division of labor

**^‡^**Colonies with adults of two-generations (matrifilial)

**^^**Female castes are morphologically different; gynes (if any) cannot survive alone

**^^**Progressive feeding

**^♦^**New colonies established by swarming
